# Determinants of population responses to environmental fluctuations

**DOI:** 10.1038/s41598-017-18976-6

**Published:** 2018-01-17

**Authors:** Jose M. G. Vilar, J. Miguel Rubi

**Affiliations:** 1 Biofisika Institute (CSIC, UPV/EHU) and Department of Biochemistry and Molecular Biology, University of the Basque Country UPV/EHU, 48080 Bilbao, Spain; 2 IKERBASQUE, Basque Foundation for Science, 48011 Bilbao, Spain; 3 Departament de Fisica de la Materia Condensada, Universitat de Barcelona, Diagonal 647, 08028 Barcelona, Spain

## Abstract

Environmental fluctuations, such as changing conditions and variable nutrient availability, are an unavoidable component of the dynamics of virtually all populations. They affect populations in ways that are often difficult to predict and sometimes lead to paradoxical outcomes. Here, we present a general analytical approach to examine how populations respond to fluctuations. We show that there exist general explicit conditions that determine to what extent fluctuations propagate to the variability of the responses and how they change the behavior of the system, including whether they promote proliferation or death and whether they facilitate coexistence or exclusion of competing species. These conditions depend on linear and nonlinear terms of the growth rate and on the characteristic times of the fluctuations. We validated our general approach through computational experiments for both stochastic and chaotic fluctuations and for multiple types of systems. From an applied point of view, our results provide an avenue for the precise control of the population behavior through fluctuations in addition to just through average properties.

## Introduction

Fluctuations are present at many levels in the dynamics of virtually all populations, extending from the inherent stochastic behavior of the individuals to the unavoidable external random perturbations^1–3^. They are fundamental determinants of phenomena as diverse as phenotype switching in changing environments^4,5^, survival in the presence of death stimuli^6,7^, and the establishment of cooperation^8^ and communities^9^. The resulting stochasticity, compounded with additional fluctuating external factors, propagates through the collective dynamics of the individuals^10^ to the large-scale population dynamics^1,11,12^.

To faithfully understand these types of systems, one needs to take into account the interplay between fluctuations and the dynamics of the population^13–15^. The effects of fluctuations, however, are often difficult to assess, especially when nonlinearities are involved, and sometimes have paradoxical outcomes. Currently, it is clear that fluctuations, often referred to as noise, are not just a source of disorder or a nuisance to be avoided. There are many known phenomena, such as stochastic resonance^16^ and multiresonance^17^, noise-induced transitions^18^, and noise suppression by noise^19^, which portray constructive roles of noise. Despite all these advances, there are still many long-standing challenges that remain unsolved^20,21^. A most pressing outstanding question is to know precisely how irregular changes in external conditions shape the population behavior and what are the determinants of their effects^20–24^.

Here, we present a wide-ranging analytical framework to analyze the effects of fluctuations on the population behavior. Using this framework, we show that there are general conditions based on the functional dependence of the growth rates and the effective correlation times of the fluctuations that determine the major outcomes of the interplay between external fluctuations and the population dynamics. Explicitly, depending on the conditions, environmental fluctuations can promote proliferation or death and facilitate coexistence or exclusion of species. We develop first a single-species framework to capture the key elements that are at play. We illustrate its wide applicability with examples covering all three classical types of functional responses for linear, concave, and convex dependences of the grow rate on the fluctuating variable. As fluctuating variables, we consider stochastic and deterministic chaotic fluctuations with exponential and linear piece-wise correlation functions, respectively. The analytical results and their applicability are exhaustively verified through computational experiments. We subsequently extend the framework to the multispecies case and study how fluctuations affect the exclusion-coexistence boundary of competing species.

The conditions we uncovered show that the value of the concavity of the growth rate with respect to the fluctuating parameter is one of the major determinants of the outcome. In general, positive values of the concavity contribute to increasing proliferation and negative values have the opposite effect. These contributions dominate the outcome for sufficiently fast fluctuations. For fluctuations with characteristic time scales similar to those of the dynamics of the system, however, the conditions show that there are other contributions that become relevant to the extent of being able to reverse the contributions of the concavity. We show that these contributions arise from effects of the resulting population variability on the dynamics of the net proliferation and that they are fundamental to provide a general predictive description of the effects of fluctuations on the population behavior.

## Results

We consider the general class of systems described by the population sizes *N*_*i*_ of a set of *M* interacting species or population types, which are represented collectively by the column vector **N** = (*N*_1_, …*N*_*M*_)^T^. The dynamics is given by general growth equations of the type

1 $$\frac{d{N}_{i}}{dt}={g}_{i}(c(t),{\bf{N}}){N}_{i},$$

with *i* = 1, … *M*. Here, *c*(*t*) represents an external quantity or environmental variable, such as nutrient concentration or temperature, that affect the per capita growth rates *g*_*i*_(*c*(*t*), **N**).

Because the external parameters are in general fluctuating quantities, we use the decomposition $c(t)\equiv \bar{c}+\varepsilon (t)$ in terms of the time-average value $\bar{c}={\langle c(t)\rangle }_{t}$ and the fluctuating contribution $\varepsilon (t)=c(t)-\bar{c}$. The fluctuation term is characterized through the correlation 〈*ε*(*t*)*ε*(*s*)〉 = *σ*^2^*f*(*t* − *s*), where *f* represents a general function with *f*(0) = 1 and finite integral ${\int }_{0}^{\infty }f(s)ds=\tau$. For instance, in the prototypical case of an exponentially correlated fluctuation term, this function is explicitly given by *f*(*t*) = *e*^−|*t*|/*τ*^. The quantities *σ*^2^ and *τ* can be interpreted as noise amplitude and correlation time, respectively. The correlation function is defined as *C*(*t*, *s*) = 〈*ε*(*t*)*ε*(*s*)〉 and therefore *f*(*t* − *s*) = *C*(*t*, *s*)/*σ*^2^ can be viewed as a normalized correlation function.

### A single-species analytic framework

The effects of fluctuations in population dynamics have been analyzed most prominently by means of simulations or analytical calculations on specific systems^25,26^. Here, to systematically study how fluctuations in the external parameters impact the behavior of the broad class of systems described by Eq. (1), we develop a novel type of closed fluctuation expansion up to the leading dominant terms of the growth rate on the amplitudes of both the environmental fluctuations and the resulting variability of the population responses.

Since population sizes typically change over several orders of magnitude, we start by considering the equivalent time evolution in terms of the logarithms of the population sizes $y=\,\mathrm{ln}\,N$,

2 $$\frac{dy}{dt}=g(c(t),{e}^{y}),$$

To perform the fluctuation expansion, we rewrite *y* as $\bar{y}+\eta$, where $\bar{y}=\langle y\rangle$ is the average of *y* over the environmental fluctuations and $\eta =y-\bar{y}$ is the remaining fluctuating term of the population, which indicates the variability of the response. Expanding *g* in *η* and *ε* up to second order in Eq. (2) and taking averages over the environmental fluctuations leads to

3 $$\frac{d}{dt}\bar{y}=g+\frac{1}{2}{\sigma }^{2}{\partial }_{cc}g+\langle \varepsilon \eta \rangle {\partial }_{cy}g+\frac{1}{2}\langle {\eta }^{2}\rangle {\partial }_{yy}g,$$

where $g\equiv g(\bar{c},{e}^{\bar{y}})$. Here, we have used that the averages of the fluctuating quantities *ε* and *η* are zero, 〈*η*〉 = 0 and 〈*ε*〉 = 0, by definition. Similarly, expanding *g* in *η* and *ε* up to first order in Eq. (1) and subtracting the first order average behavior leads to

4 $$\frac{d}{dt}\eta =\varepsilon {\partial }_{c}g+\eta {\partial }_{y}g.$$

The initial conditions are $\bar{y}(0)=y(0)$ and *η*(0) = 0 when the population size is specified at the initial time.

We obtain the dynamics of 〈*η*^2^〉 from Eq. (4) by making use of the identity *d*〈*η*^2^〉/*dt* = 2〈*ηdη*/*dt*〉. As explicit expression of *dη*/*dt*, we use the right-hand side of Eq. (4), which leads to

5 $$\frac{d\langle {\eta }^{2}\rangle }{dt}=2\langle \varepsilon \eta \rangle {\partial }_{c}g+2\langle {\eta }^{2}\rangle {\partial }_{y}g,$$

with initial condition 〈*η*^2^〉(0) = 0.

The cross-correlation term 〈*εη*〉 is computed from the formal solution of Eq. (4) considering that *ε* changes in time substantially faster than ∂_*y*_*g* and ∂_*c*_ *g*,

6 $$\eta (t)={\int }_{0}^{t}({\partial }_{c}\,g){e}^{({\partial }_{y}g)(t-s)}\varepsilon (s)ds,$$

which after multiplication by *ε*(*t*) and averaging over the fluctuations results in

7 $$\langle \varepsilon \eta \rangle =({{\rm{\partial }}}_{c}\,g){\sigma }^{2}{\int }_{0}^{t}{e}^{({{\rm{\partial }}}_{y}g)(t-s)}f(t-s)ds.$$

Our central result for the single-species case is the closed set of Eqs (3), (5), and (7), which describe the effects of the environmental fluctuations on the dynamics of the population in terms of the average behavior, its variability, and the amplitude and correlation of the fluctuations.

There are important conclusions that can be drawn from the explicit form of Eqs (3), (5) and (7). Equation (3) specifies that the value of the concavity of the growth rate, ∂_*cc* _*g*, is one of the major determinants of the net growth rate, $d\bar{y}/dt$, with positive values contributing to its increase and negative values contributing to its decrease. Equations (5) and (7) show that the population variability, 〈*η*^2^〉, increases with the square of the linear dependence of the growth rate, (∂_*c*_ *g*)^2^. For small correlation times of the fluctuations, the integral in Eq. (7) can be approximated as ${\int }_{0}^{t}{e}^{({\partial }_{y}g)(t-s)}f(t-s)ds\approx \tau$ and therefore Eqs (5) and (7) imply that the population variability is also an increasing function of the correlation time *τ*. These results indicate that, in the limit of very fast fluctuations or very small linear dependence of the growth rate, the terms 〈*εη*〉 and 〈*η*^2^〉 become negligible in Eq. (3) and the effects of fluctuations are completely determined by the concavity of the growth rate. This decisive dependence on ∂_*cc*_ *g* is also present when the per capita growth rate does not depend on the population size, as in the case of exponential growth, because ∂_*cy*_ *g* and ∂_*yy*_ *g* are zero under these conditions. In general, however, the effects of the fluctuations on the population behavior depend on the multiple terms present in Eqs (3), (5), and (7). These terms take into account the effects of the population variability in the net growth rate and have traditionally been overlooked in other approaches^23,26^ based on the small noise expansion technique^27,28^.

### Multiple types of environmental fluctuations

The cross-correlation of the environmental fluctuations with the population variability 〈*εη*〉 can be computed explicitly from Eq. (7) for specific forms of the correlation function. The archetypical cases involve exponentially correlated fluctuations, 〈*ε*(*t*)*ε*(*s*)〉 = *σ*^2^*e*^−|*t*−*s*|/*τ*^, which lead to

8 $$\langle \varepsilon \eta \rangle =\frac{{\partial }_{c}g{\sigma }^{2}}{{\partial }_{y}g-{\tau }^{-1}}(1-{e}^{({\partial }_{y}g-{\tau }^{-1})t}).$$

Another important type of correlation function is the triangular correlation $$\langle \varepsilon (t)\varepsilon (s)\rangle ={\sigma }^{2}\,\max $$$$(1-|t-s|/(2\tau ),\,0)$$, which leads to

9 $$\langle \varepsilon \eta \rangle ={\partial }_{c}\,g{\sigma }^{2}\{\begin{array}{c}\frac{{e}^{{\partial }_{y}gt}(1+{\partial }_{y}g(2\tau -t))-(1+{\partial }_{y}g2\tau )}{{({\partial }_{y}g)}^{2}2\tau }\,if\,t < 2\tau \\ \frac{{e}^{{\partial }_{y}g2\tau }-(1+{\partial }_{y}g2\tau )}{{({\partial }_{y}g)}^{2}2\tau }\,if\,t\ge 2\tau \end{array}$$

This type of correlation function arises, for instance, in the deterministic time series generated with a chaotic logistic map^29^.

In general, when ∂_*y*_*g* is a negative quantity, as required by stability considerations, the two factors in the integrand of Eq. (7) are both smaller than one and we have

10 $$0\le |\langle \varepsilon \eta \rangle | < {\sigma }^{2}|{{\rm{\partial }}}_{c}\,g|min(-1/{{\rm{\partial }}}_{y}g,\tau ).$$

This expression implies that 〈*εη*〉 ≈ 0 and, through Eq. (5), that 〈*η*^2^〉 ≈ 0 when the dynamics of the fluctuations are very fast or when the system is very stable.

### Hierarchical control of proliferation

Equations (3), (5) and (7) provide an avenue to compute the effects of the environmental fluctuations on the growth rate, which we quantify through

11 $${\rm{\Delta }}\bar{g}=d\bar{y}/dt-g.$$

Therefore, the net growth rate increases when ${\rm{\Delta }}\bar{g} > 0$ and decreases when ${\rm{\Delta }}\bar{g} < 0$. If ${\rm{\Delta }}\bar{g}=0$, the average growth rate remains unaltered.

*Initial time* - At time zero, if the population is specified, we have 〈*εη*〉 = 0 and 〈*η*^2^〉 = 0. Therefore, the value of ${\rm{\Delta }}\bar{g}$ that determines how environmental fluctuations affect growth is

12 $${\rm{\Delta }}{\bar{g}}_{I}=\frac{1}{2}({\partial }_{cc}\,g){\sigma }^{2},$$

which follows straightforwardly from Eq. (3). The value of this quantity depends on the concavity, an inherently non-linear property, of the growth rate with respect to the fluctuating parameter and on the amplitude of the fluctuations and it can take positive and negative values^30^.

*Early stages* - For intermediate times of the order of *τ*, the term 〈*εη*〉 is generally different from zero and dominates over 〈*η*^2^〉, which leads to a more complex expression of ${\rm{\Delta }}\bar{g}$ given by

13 $${\rm{\Delta }}{\bar{g}}_{E}=(\frac{1}{2}{\partial }_{cc}g+{\partial }_{cy}g{\partial }_{c}g\tilde{\tau }){\sigma }^{2},$$

where we have used the notation $\tilde{\tau }\equiv {\int }_{0}^{t}{e}^{({\partial }_{y}g)(t-s)}f(t-s)ds$. In this case, not just the amplitude of the fluctuations but also their correlation is important. At this stage, linear terms, such as the slope of the growth rate with respect to the fluctuating parameter, become relevant.

*Late stages* - For longer times, if the system reaches a steady state, we have from Eq. (5) that 〈*η*^2^〉 = −(∂_*c*_ *g*)/(∂_*y*_*g*)〈*εη*〉. Therefore, the value of ${\rm{\Delta }}\bar{g}$ that determines the effects of fluctuations in the growth rate is

14 $${\rm{\Delta }}{\bar{g}}_{L}=[\frac{1}{2}{\partial }_{cc}g+({\partial }_{cy}g-\frac{1}{2}{\partial }_{yy}g\frac{{\partial }_{c}g}{{\partial }_{y}g}){\partial }_{c}g\tilde{\tau }]{\sigma }^{2}.$$

This quantity shows a complex dependence on multiple properties of the growth rate, which account for the effects of both environmental fluctuations and the feedback of the resulting population variability.

It is important to emphasize that the value of the concavity of the growth rate with respect to the fluctuating parameter is one of the major determinants of the sign of equations. This contribution dominates the outcome for sufficiently fast fluctuations but there are other terms in the equations that might become relevant for fluctuations with characteristic time scales similar to those of the dynamics of the system.

### General applicability

To illustrate explicitly the general applicability of our results, we consider three prototypical dependences of the growth rate on the population size, namely, exponential growth,

15 $$g(c,N)=r(c)-\gamma ,$$

growth with saturation,

16 $$g(c,N)=r(c)/(1+N)-\gamma ,$$

and logistic growth,

17 $$g(c,N)=r(c)(1-N)-\gamma ,$$

where *γ* is a constant and *r*(*c*) is a function of the fluctuating parameter.

For exponential growth, we have ∂_*c*_ *g* = *r*′, ∂_*y*_*g* = 0, ∂_*cc*_ *g* = *r*′′, ∂_*cy*_ *g* = 0, and ∂_*yy*_ *g* = 0, which leads to

18 $${\rm{\Delta }}{\bar{g}}_{I}={\rm{\Delta }}{\bar{g}}_{E}={\rm{\Delta }}{\bar{g}}_{L}=\frac{1}{2}r^{\prime\prime} {\sigma }^{2}.$$

These results indicate that the effects of environmental fluctuations on the average behavior are time-invariant and do not depend on the correlation time.

For growth with saturation, the explicit values ${\partial }_{c}g=r^{\prime} {(1+{e}^{\bar{y}})}^{-1}$, ${\partial }_{y}g=-r{(1+{e}^{\bar{y}})}^{-2}$, ${\partial }_{cc}g=r^{\prime\prime} {(1+{e}^{\bar{y}})}^{-1}$, ${\partial }_{cy}g=-r^{\prime} {(1+{e}^{\bar{y}})}^{-2}$, and ${\partial }_{yy}g=2r{(1+{e}^{\bar{y}})}^{-3}$ lead to

19 $$\begin{array}{c}{\rm{\Delta }}{\bar{g}}_{I}=\frac{1}{2}r^{\prime\prime} {(1+{e}^{\bar{y}})}^{-1}{\sigma }^{2}\\ {\rm{\Delta }}{\bar{g}}_{E}=\frac{1}{2}[r^{\prime\prime} -{(1+{e}^{\bar{y}})}^{-2}{(r^{\prime} )}^{2}\tilde{\tau }]{(1+{e}^{\bar{y}})}^{-1}{\sigma }^{2}\\ {\rm{\Delta }}{\bar{g}}_{L}=\frac{1}{2}r^{\prime\prime} {(1+{e}^{\bar{y}})}^{-1}{\sigma }^{2}.\end{array}$$

In this case, the correlation time of the fluctuations affects the early stages of the average growth but the specific form of the different derivatives leads to a cancelation of this dependence at the late stages.

For logistic growth, we have ${\partial }_{c}g=r^{\prime} (1-{e}^{\bar{y}})$, ${\partial }_{y}g=-r{e}^{\bar{y}}$, ${\partial }_{cc}g=r^{\prime\prime} (1-{e}^{\bar{y}})$, ${\partial }_{cy}g=-r^{\prime} {e}^{\bar{y}}$, and ${\partial }_{yy}g=r{e}^{\bar{y}}$, which results in

20 $$\begin{array}{c}{\rm{\Delta }}{\bar{g}}_{I}=\frac{1}{2}r^{\prime\prime} (1-{e}^{\bar{y}}){\sigma }^{2}\\ {\rm{\Delta }}{\bar{g}}_{E}=[\frac{1}{2}r^{\prime\prime} -{e}^{\bar{y}}{(r^{\prime} )}^{2}\tilde{\tau }](1-{e}^{\bar{y}}){\sigma }^{2}\\ {\rm{\Delta }}{\bar{g}}_{L}=[\frac{1}{2}r^{\prime\prime} -\frac{1}{2}(3{e}^{\bar{y}}-1){(r^{\prime} )}^{2}\tilde{\tau }](1-{e}^{\bar{y}}){\sigma }^{2}.\end{array}$$

In this case, the correlation time of the fluctuations affects both the early and late stages of the average growth. It is important to emphasize that if *r*′′ is positive, increasing the correlation time can potentially change the effect of the environmental fluctuations from enhancing to suppressing the average growth rate.

Our analysis of these three representative examples indicates that environmental fluctuations can affect the net proliferation of populations in multiple ways, leading to both positive and negative changes in the growth rate. These changes, which depend on multiple properties of the system, as described by Eq. (14), do not necessarily remain fixed in time but can evolve and can even potentially switch between positive and negative values as time progresses.

### Computational experiments

**Figure 1:**
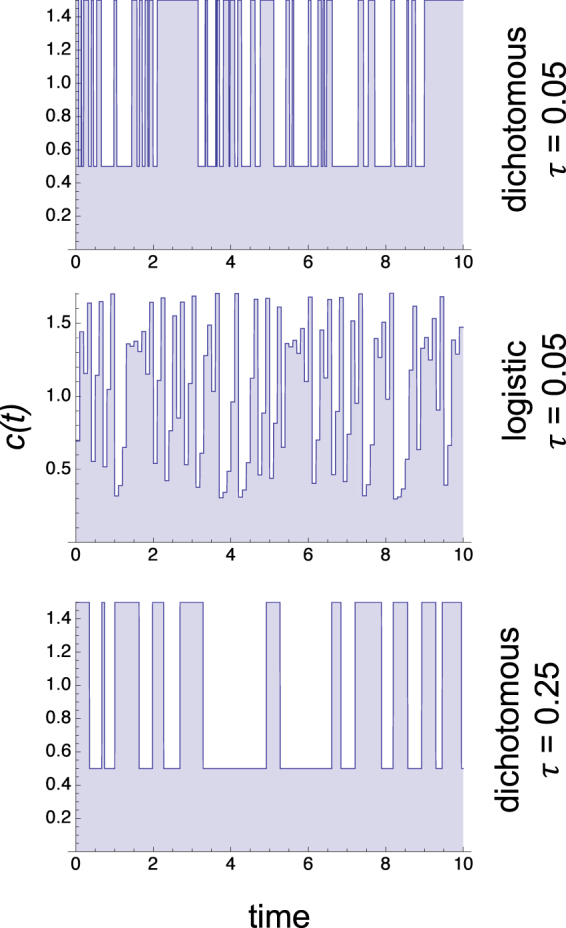
Representative types of environmental fluctuations. The typical time courses of the fluctuating parameter $c=\bar{c}+\varepsilon (t)$ are shown for the dichotomous random process (Eq. (35)) with *σ* = 0.5, $\bar{c}=1$, and correlation times *τ* = 0.05 and *τ* = 0.25, as shown in the panels, and for the chaotic logistic map (Eqs (36) and (37)) with *σ* = 0.5, $\bar{c}=1$, and correlation time *τ* = 0.05.

To illustrate the wide applicability of our results and to validate the predictions of our analysis, we performed computational experiments for two radically different types of fluctuations and multiple dependences of the growth rate on the fluctuating parameter. As explicit examples of environmental fluctuations, we consider the dichotomous random process with transitions given by Eq. (35) and the deterministic time series generated with a chaotic logistic map given by Eqs (36) and (37), which are detailed in Materials and Methods. The dichotomous random process, also known as telegraph process^31^, is the prototypical case of exponentially correlated random process and the chaotic logistic map is a deterministic system with irregular behavior that can be characterized in terms of probabilities^29^. Both of these types of fluctuations lead to highly irregular changes in the parameter *c*(*t*), as illustrated in Fig. 1.

We consider three specific forms of *r*(*c*) in Eqs (15), (16), and (17) to cover three representative scenarios for the effects of external fluctuations with three different values of the second derivative of the growth rates with respect to the fluctuating parameter:

21 $$r(c)={r}_{0}c/{c}_{0}\,$$

for zero values,

22 $$r(c)=2{r}_{0}\frac{c/{c}_{0}}{1+c/{c}_{0}}$$

for negative values, and

23 $$r(c)={r}_{0}{(c/{c}_{0})}^{2}\,$$

for positive values. In these cases, *c* is an external parameter, such as the concentration of nutrients or prey density, *c*_0_ is a characteristic value, and *r*_0_ is the growth rate at *c* = *c*_0_^32^. In ecology, the general forms of *r*(*c*) in Eqs (21) and (22) correspond to prototypical examples of Holling’s type I and II functional responses, respectively, and that in Eq. (23) corresponds to a type III functional response in the low nutrient density regime^33^.

**Figure 2:**
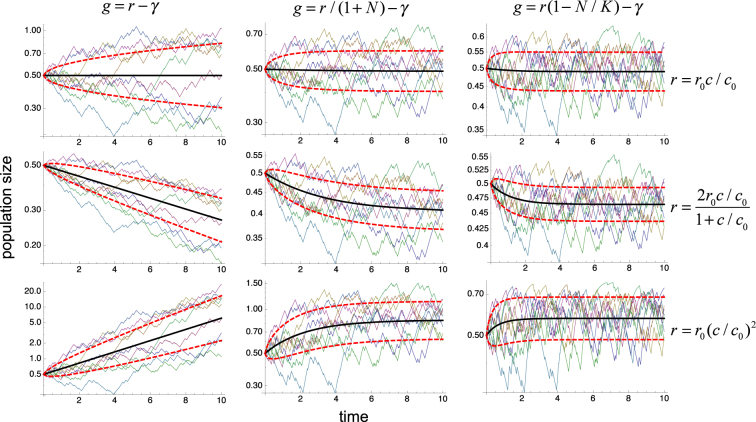
Control of proliferation by random fluctuations. The typical time courses of the populations described by Eq. (2) are shown for growth rates given by all the combinations of Eqs (21), (22) and (23) with Eqs (15), (16) and (17) and for fluctuating parameter $c=\bar{c}+\varepsilon (t)$. The thin lines correspond to different realizations of the random fluctuations, which are the same for all panels. The external fluctuations *ε*(*t*) follow a dichotomous random process with zero mean, standard deviation *σ* = 0.5, and correlation time *τ* = 0.05. The values of the remaining parameters are *γ* = 1, *r*_0_ = 1, $\bar{c}=1$, and *c*_0_ = 1. The initial condition is *N* = 0.5. The continuous thick line corresponds to the average behavior ${e}^{\bar{y}}$ given by Eq. (3) and the dashed thick lines correspond to ${e}^{\bar{y}+\sqrt{\langle {\eta }^{2}\rangle }}$ and ${e}^{\bar{y}-\sqrt{\langle {\eta }^{2}\rangle }}$ from Eqs (3) and (5).

The results of simulating the nine cases resulting from all the possible combinations of Eqs (21), (22) and (23) with Eqs (15), (16) and (17) for different realizations of the dichotomous random process are illustrated in Fig. 2 (thin lines). They show that the effects of fluctuations on the population’s growth are determined in all cases by the initial-, early-, and late-stage conditions provided by Eqs (18), (19) and (20). In these cases, the effects are dominated by the sign of the second derivative of *r*(*c*), with positive values promoting proliferation, negative values increasing death, and the zero value leaving the average growth rate basically unaltered. In all the cases, the individual stochastic time courses closely follow the temporal evolution of the net proliferation obtained from Eq. (3) with a spread determined by the variability predicted by Eq. (5), as shown in Fig. 2 (thick lines).

**Figure 3:**
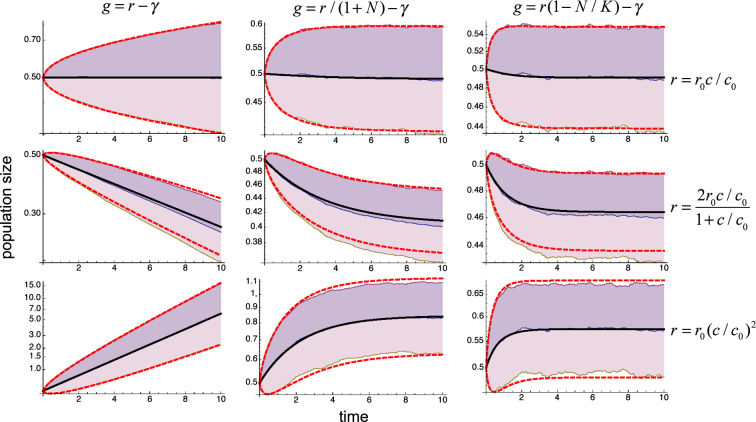
Accuracy of the analytical framework. Values of ${e}^{\bar{y}+\sqrt{\langle {\eta }^{2}\rangle }}$, ${e}^{\bar{y}}$, and ${e}^{\bar{y}-\sqrt{\langle {\eta }^{2}\rangle }}$ computed over 1,000 realizations of the dichotomous process (shaded regions) are compared with the results from Eqs (3) and (5) (thick lines) for the same systems and conditions as in Fig. 2.

To verify in detail the accuracy of the predictions of Eqs (3) and (5), we performed computer simulations for 1,000 different realizations of the dichotomous random process and computed the average and variance of *y* over all of them along the temporal evolution. The results from the analytical framework (thick lines) and computational experiments (shaded regions) show an exceptional agreement with each other, as illustrated in Fig. 3.

**Figure 4:**
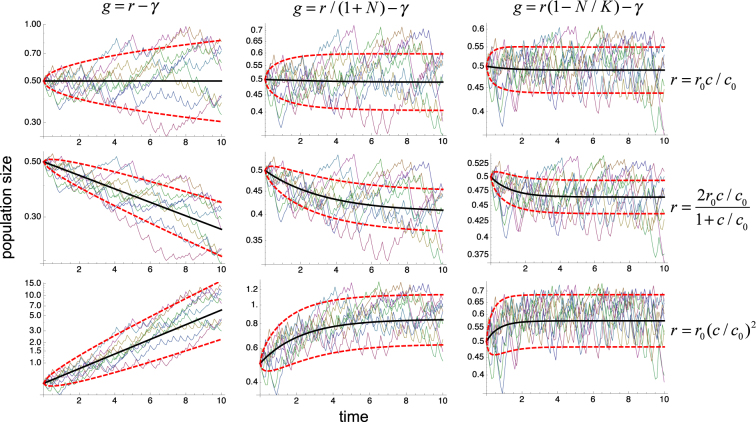
Control of proliferation by deterministic chaotic fluctuations. The typical time courses of the populations described by Eq. (2) are shown for the same systems and conditions as in Fig. 2 except that the external fluctuations *ε*(*t*) are generated by deterministic chaotic fluctuations from the logistic map (Eqs (36) and (37)) with *σ* = 0.5, $\bar{c}=1$, and correlation time *τ* = 0.05. Different time courses correspond to different initial conditions in Eq. (36).

Similarly, as in the dichotomous random process, the initial-, early-, and late-stage conditions specified by Eqs (18), (19) and (20) also dictate the sign of the effects of the determinist chaotic fluctuations described by Eqs (36) and (37), as illustrated in Fig. 4 (thin lines). In this case as well, the spread of the responses closely follows the variability predicted by Eq. (5), as shown in Fig. 4 (thick lines).

**Figure 5:**
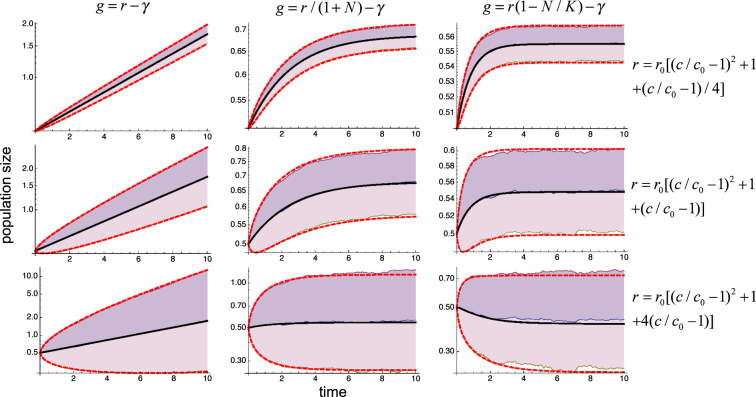
Effects of linear terms. Values of ${e}^{\bar{y}+\sqrt{\langle {\eta }^{2}\rangle }}$, ${e}^{\bar{y}}$, and ${e}^{\bar{y}-\sqrt{\langle {\eta }^{2}\rangle }}$ computed over 1,000 realizations of the dichotomous process (shaded regions) are compared with the results from Eqs (3) and (5) (thick lines) for growth rates given by all the combinations of Eqs (21), (22) and (23) with three different values of the linear term in Eq. (24). The fluctuating parameter $c=\bar{c}+\varepsilon (t)$ has external fluctuations *ε*(*t*) generated with a dichotomous random process with zero mean, standard deviation *σ* = 0.5, and correlation time *τ* = 0.05. The values of the remaining parameters are *γ* = 1, *r*_0_ = 1, and *c*_0_ = 1. The initial condition is *N* = 0.5.

Our general analytical results (Eqs (3), (5) and (7)) and their application to explicit systems (Eqs (18), (19) and (20)) indicate that the linear dependence of *g* on *c* through ∂_*c*_ *g* and ∂_*cy*_ *g* plays a fundamental role in both the net proliferation and the variability of the responses. To analyze these effects in detail, we consider

24 $$r(c)={r}_{0}[{(c/{c}_{0}-1)}^{2}+1+\alpha (c/{c}_{0}-1)]$$

in Eqs (15), (16) and (17). We performed simulations for 1,000 different realizations of the dichotomous random process and computed the average and variance of *y* over all of them along the temporal evolution for different values *α*. The results show that indeed increasing the linear term increases the variability and strongly affects the net proliferation rate, to the extent of changing the effects from promoting to suppressing growth, as illustrated in Fig. 5. The agreement between theory and computational experiments is exceptional, even for large variability of the responses, of about a factor three.

**Figure 6:**
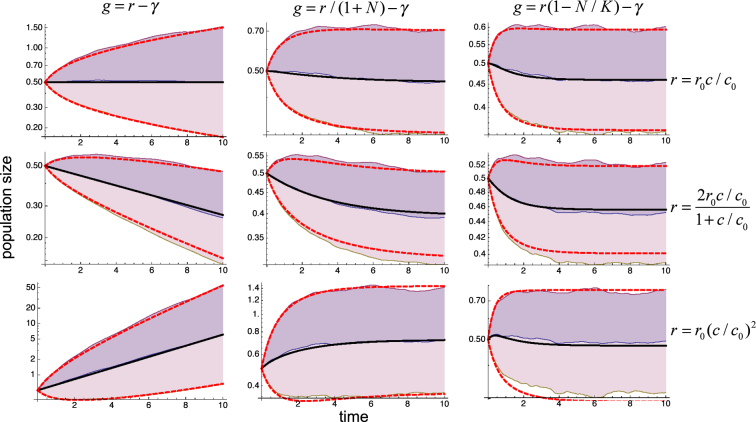
Effects of the fluctuations correlation time. Values of ${e}^{\bar{y}+\sqrt{\langle {\eta }^{2}\rangle }}$, ${e}^{\bar{y}}$, and ${e}^{\bar{y}-\sqrt{\langle {\eta }^{2}\rangle }}$ computed over 1,000 realizations of the dichotomous process (shaded regions) are compared with the results from Eqs (3) and (5) (thick lines), as in Fig. 3, for the same systems and conditions as in Figs 2 and 3 except that the correlation time has been increased by a factor 5 to *τ* = 0.25.

Our results also show that increasing the correlation time consistently leads to higher variability of the responses. The effects on the net proliferation rate, however, are more intricate. To illustrate these effects, we show in Fig. 6 the results obtained using the same systems as in Fig. 3 but with a correlation time 5 times bigger. In the cases described by Eqs (15) and (16), increasing the correlation time has no significant effect on the average growth rate. In the case described by Eq. (17), it suppresses growth to the extent of overcoming the positive effects of *r*′′ > 0. In all the cases, the results of the computational experiments show an excellent agreement with the theory.

### A multi-species analytic framework

To extend our results to the multi-species case, we proceed as in the analysis of Eq. (2) but using matrix calculus. We consider the logarithms of the population sizes ${y}_{i}=\,\mathrm{ln}\,{N}_{i}$, or equivalently ${\bf{y}}={(\mathrm{ln}{N}_{1},\mathrm{...}\mathrm{ln}{N}_{M})}^{{\rm{T}}}$, which leads to

25 $$\frac{d{\bf{y}}}{dt}={\bf{g}}(c(t),{{\bf{e}}}^{{\bf{y}}}),$$

where ${{\bf{e}}}^{{\bf{y}}}={({e}^{{y}_{1}},\mathrm{...}{e}^{{y}_{M}})}^{{\rm{T}}}={\bf{N}}$ and **g** = (*g*_1_, … *g*_*M*_)^T^. As in the single-species case, we rewrite ***y*** as $\bar{{\bf{y}}}{\boldsymbol{+}}{\boldsymbol{\eta }}$, where $\bar{{\bf{y}}}=\langle {\bf{y}}\rangle$ is the average of ***y*** over the environmental fluctuations and ${\boldsymbol{\eta }}={\bf{y}}-\bar{{\bf{y}}}$ is the remaining fluctuating term. Expanding **g** in **η** and *ε* up to second order in Eq. (25) and taking averages over the environmental fluctuations leads to

26 $$\frac{d}{dt}\bar{{\bf{y}}}={\bf{g}}+\frac{1}{2}{{\rm{\partial }}}_{c,c}{\bf{g}}{{\boldsymbol{\sigma }}}^{2}+({\langle \varepsilon {\boldsymbol{\eta }}\rangle }^{{\rm{T}}}{{\rm{\partial }}}_{{\bf{y}},c}){\bf{g}}+\frac{1}{2}(\langle {\boldsymbol{\eta }}{{\boldsymbol{\eta }}}^{{\rm{T}}}\rangle {{\rm{\partial }}}_{{y,y}^{{\rm{T}}}}){\bf{g}},$$

with ${\bf{g}}\equiv {\bf{g}}(c,{{\bf{e}}}^{\bar{{\bf{y}}}})$. Consequently, the dynamics of the fluctuating term is given by

27 $$\frac{d}{dt}{\boldsymbol{\eta }}={\partial }_{c}{\bf{g}}\varepsilon +{({\partial }_{{\rm{y}}}{{\bf{g}}}^{{\rm{T}}})}^{{\rm{T}}}{\boldsymbol{\eta }}{\boldsymbol{,}}$$

where we have used (**η**^T^∂_***y***_)***g*** = (((**η**^T^∂_***y***_)***g***)^T^)^T^ = (**η**^T^∂_***y***_***g***^T^)^T^ = (∂_***y***_***g***^T^)^T^**η**. The initial conditions are $\bar{{\bf{y}}}(0)={\bf{y}}(0)$ and **η**(0) = **0**.

We obtain the dynamics of 〈**ηη**^T^〉 from Eq. (27) making use of the identity *d*〈**ηη**^T^〉/*dt* = 2〈(*d***η**/*dt*)**η**^T^〉, which leads to

28 $$\frac{d}{dt}\langle {\boldsymbol{\eta }}{{\boldsymbol{\eta }}}^{{\rm{T}}}\rangle =2{\partial }_{c}{\bf{g}}\langle \varepsilon {{\boldsymbol{\eta }}}^{{\rm{T}}}\rangle +2{({\partial }_{{\bf{y}}}{{\bf{g}}}^{{\rm{T}}})}^{{\rm{T}}}\langle {\boldsymbol{\eta }}{{\boldsymbol{\eta }}}^{{\rm{T}}}\rangle .$$

Analogously to the single species case, the formal solution of Eq. (27) is

29 $${\boldsymbol{\eta }}(t)={\int }_{0}^{t}{e}^{{({\partial }_{{\bf{y}}}{{\bf{g}}}^{{\rm{T}}})}^{{\rm{T}}}(t-s)}{\partial }_{c}{\bf{g}}\varepsilon (s)ds,$$

which leads to

30 $$\langle \varepsilon {\boldsymbol{\eta }}\rangle ={\int }_{0}^{t}{e}^{{({\partial }_{{\bf{y}}}{{\bf{g}}}^{{\rm{T}}})}^{{\rm{T}}}(t-s)}{\partial }_{c}{\bf{g}}\langle \varepsilon (s)\varepsilon (t)\rangle ds.$$

Our central result for the multiple-species case is the closed set of Eqs (26), (28) and (30), which provides the multispecies counter part of Eqs (3), (5) and (7).

### Control of coexistence

There are scenarios where the effects of external fluctuations can be subtler than just promoting proliferation or death but with more dramatic consequences. In the prototypical case of symmetric two-species competition^34^ with per capita growth rates

31 $${\bf{g}}=(\begin{array}{c}{r}_{0}(1-{N}_{1}-\beta {N}_{2})\\ {r}_{0}(1-{N}_{2}-\beta {N}_{1})\end{array})=(\begin{array}{c}{r}_{0}(1-{e}^{{y}_{1}}-\beta {e}^{{y}_{2}})\\ {r}_{0}(1-{e}^{{y}_{2}}-\beta {e}^{{y}_{1}})\end{array}),$$

the stability of the coexisting state depends of the coupling parameter *β*. The stability analysis of the deterministic dynamics indicates that both species coexist when *β* ≤ 1 and that one species excludes the other when *β* > 1. However, when *β* is a function of a fluctuating external parameter *c*, such as a common resource upon which both species feed, the results of the stability analysis are no longer applicable and it is not clear a priori whether both species can coexist or one of them excludes the other.

Our approach, through the use of Eqs (26), (28) and (30), provides the effective dynamics for the average of the logarithm of respective population sizes as

32 $$\frac{d}{dt}\bar{{\bf{y}}}={\bf{g}}+\frac{1}{2}{\partial }_{c,c}{\bf{g}}{\sigma }^{2}+({\partial }_{c}{{\bf{g}}}^{{\rm{T}}}{\partial }_{{\bf{y}},c}){\bf{g}}{\sigma }^{2}\tau $$

where we have assumed short correlation times to simplify Eq. (30) into 〈*ε***η**〉 ≈ ∂_*c*_**g***σ*^2^*τ* and early stages so that the term $\frac{1}{2}(\langle {\boldsymbol{\eta }}{{\boldsymbol{\eta }}}^{{\rm{T}}}\rangle {\partial }_{{\bf{y}},{{\bf{y}}}^{{\rm{T}}}}){\bf{g}}$ can be neglected.

The values of the derivatives are given by

33 $$\begin{array}{cc}{\partial }_{c}{\bf{g}}=-{r}_{0}(\begin{array}{c}\beta ^{\prime} {e}^{{\bar{y}}_{2}}\\ \beta ^{\prime} {e}^{{\bar{y}}_{1}}\end{array}), & ({\partial }_{c}{{\bf{g}}}^{{\rm{T}}}{\partial }_{{\bf{y}},c}){\bf{g}}={({r}_{0}\beta ^{\prime} )}^{2}(\begin{array}{c}{e}^{{\bar{y}}_{1}}{e}^{{\bar{y}}_{2}}\\ {e}^{{\bar{y}}_{2}}{e}^{{\bar{y}}_{1}}\end{array}),\end{array}$$

which lead to

34 $$\frac{d}{dt}(\begin{array}{c}{\bar{y}}_{1}\\ {\bar{y}}_{2}\end{array})=(\begin{array}{c}{r}_{0}[1-{e}^{{\bar{y}}_{1}}-({\beta }_{0}+\beta ^{\prime\prime} {\sigma }^{2}/2){e}^{{\bar{y}}_{2}}]+{({r}_{0}\beta ^{\prime} )}^{2}{e}^{{\bar{y}}_{1}}{e}^{{\bar{y}}_{2}}{\sigma }^{2}\tau \\ {r}_{0}[1-{e}^{{\bar{y}}_{2}}-({\beta }_{0}+\beta ^{\prime\prime} {\sigma }^{2}/2){e}^{{\bar{y}}_{1}}]+{({r}_{0}\beta ^{\prime} )}^{2}{e}^{{\bar{y}}_{1}}{e}^{{\bar{y}}_{2}}{\sigma }^{2}\tau \end{array}),$$

where $${\beta }_{0}=\beta (\bar{c})$$, $$\beta ={\partial \beta /\partial c|}_{c=\bar{c}}$$, and $$\beta ^{\prime\prime} ={{\partial }^{2}\beta /\partial {c}^{2}|}_{c=\bar{c}}$$.

**Figure 7:**
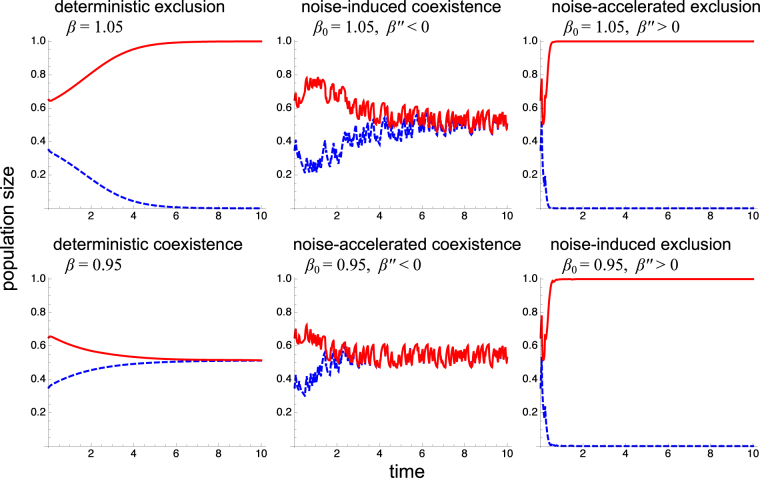
Control of coexistence by fluctuations. The typical time courses of the symmetric two-species competition dynamics described by Eq. (31) are shown for different forms of the coupling between species *β* as a function of the fluctuating parameter $c=\bar{c}+\varepsilon (t)$, which from left to right panels are given by *β*(*c*) = *β*_0_, *β*(*c*) = *β*_0_2*c*/(1 + *c*), and *β*(*c*) = *β*_0_*c*^2^ with *β*_0_ = 1.05 for the top panels and *β*_0_ = 0.95 for bottom panels. The external fluctuations *ε*(*t*) follow a dichotomous random process with zero mean, standard deviation *σ* = 0.5, and correlation time *τ* = 0.025. The value of the remaining parameter is *r*_0_ = 20.

The straightforward stability analysis of these equations indicates that the boundary of coexistence-exclusion is given by *β*_0_ + *β*′′*σ*^2^/2 = 1. Therefore, fluctuations promote coexistence if *β*′′ < 0 and exclusion if *β*′′ > 0. For values of *β*_0_ close to 1, the presence of fluctuations can even move the population from exclusion to coexistence and vice versa depending on the explicit value of *β*″, as illustrated in Fig. 7.

## Discussion

The way fluctuations shape the population behavior is an outstanding question of practical importance in fields as diverse as ecology^26^, microbiology^35^, epidemiology^36^, and economics^37^. Our results provide an analytical framework to examine the effects of external fluctuations in a wide variety of systems. Through this framework, we have obtained explicit conditions that determine to what extent fluctuations propagate to the variability of populations and how they affect fundamental properties of the system, including whether they promote or prevent proliferation and whether they stabilize or destabilize coexistence. The wide-ranging applicability of these general conditions has been extensively validated explicitly through computational experiments of single-species and multispecies dynamics, encompassing the three classical types of functional responses as well as exponential growth, growth with saturation, and logistic growth.

We found that, in general, fluctuations can both positively and negatively impact population proliferation and coexistence, depending on their precise interplay with the linear and nonlinear terms of the system. Explicitly, we found that the concavity of the per capita growth function with respect to the fluctuating parameter is a major determinant of the net proliferation whereas the linear terms regulate the variability of the responses. For sufficiently fast fluctuations, the value of the concavity decisively determines the outcomes. For fluctuations with characteristic time scales similar to those of the dynamics of the system, however, our approach uncovered that there is a significant feedback that makes the resulting population variability enter explicitly in the governing dynamics of the net proliferation. This fundamental coupling is responsible for a more complex relationship among the properties of the system in determining the effects of fluctuations. Earlier studies did not account for this type of feedback^23,26^, which prevented them from providing a general predictive description of the effects of fluctuations as the one we have presented here.

From an applied point of view, the explicit expressions we have obtained and their potential extensions provide a much-needed guiding tool to efficiently influence population systems through the properties of fluctuating quantities. In this way, our results make it possible to informedly target the quantities and the fluctuation properties that best can be used to change the behavior of populations, thus opening an avenue for controlling populations through fluctuations in addition to just through average properties.

## Materials and Methods

The dichotomous random process is based on the transitions

35 $$\begin{array}{c}{c}_{1}\mathop{\longrightarrow }\limits^{1/2\tau }{c}_{2}\\ {c}_{2}\mathop{\longrightarrow }\limits^{1/2\tau }{c}_{1}\end{array}$$

between two values *c*_1_ and *c*_2_ of *c*(*t*) with rates (2*τ*)^−1^. The mean, variance, and normalized correlation function are given by $\bar{c}=({c}_{1}+{c}_{2})/2$, *σ*^2^ = (*c*_1_ − *c*_2_)^2^/4, and *f*(*t*) = *e*^−|*t*|/*τ*^, respectively^31^. For the computational experiments, we consider exact realizations of this random process obtained with the Doob-Gillespie algorithm^38,39^.

The chaotic logistic map is described by the discrete deterministic equation

36 $${x}_{n+1}=4{x}_{n}(1-{x}_{n}),$$

which leads to the fluctuating time series

37 $$c(t)={c}_{0}+\sigma ({x}_{{\rm{floor}}(t/2\tau )}-0.5)/\sqrt{2}.$$

The floor function, which gives the greatest integer not exceeding its argument, in the subscript of *x* selects an index that increases one unit at time intervals of 2*τ*. The mean, variance, and normalized correlation function are given by $\bar{c}={c}_{0}$, *σ*^2^, and $f(t)=\,{\rm{\max }}(1-|t|/(2\tau ),0)$, respectively^29^.

The time courses of the populations are obtained by numerically integrating Eqs (2) and (25) with the corresponding growth functions for the different realizations of the fluctuations *c*(*t*). The numerical integrator used is a predictor-corrector Adams method with orders 1 through 12 ^40^.
